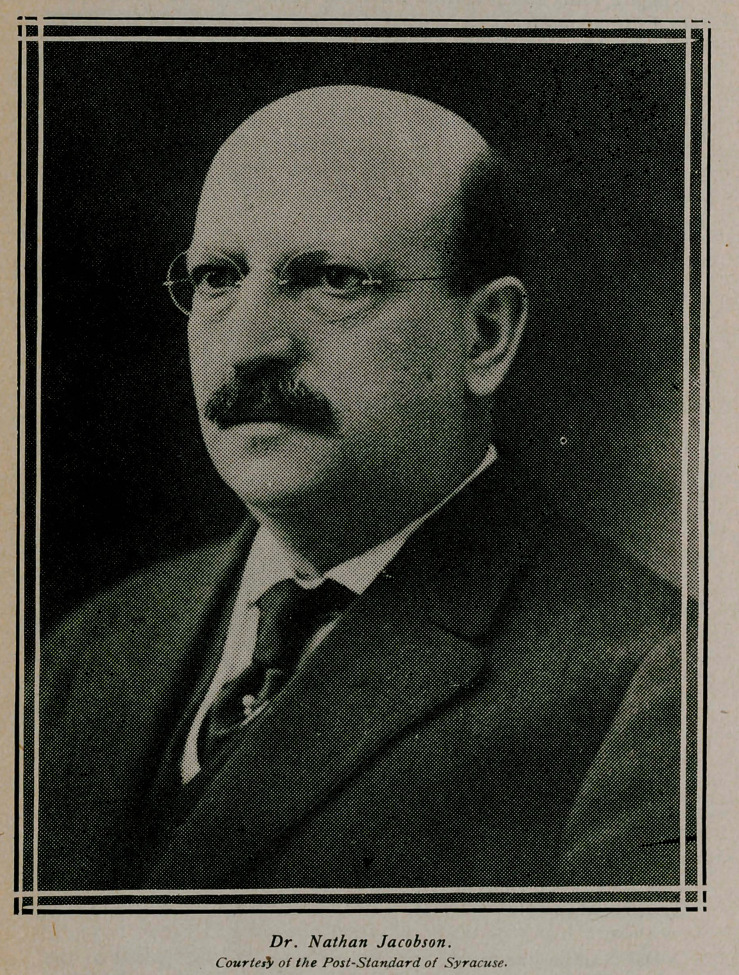# Dr. Nathan Jacobson

**Published:** 1913-10

**Authors:** 


Dr. Nathan Jacobson, Syracuse University, 1877, died at the Syracuse Hospital for Women and Children, September 16, while visiting his patients. Death was due to angina pectoris and occurred about two hours after his first seizure. There had apparently been no premonitory symptoms, as Dr. Jacobson had never mentioned them and he had remarked, just before the seizure, that he had never felt better in his life.

*Dr. Nathan Jacobson.*

*Courtesy of the Post-Standard of Syracuse.*

Dr. Jacobson was born in Syracuse, June 26, 1857, and received his education in the public schools of that city, graduating from the High School in 1874. He began the study of medicine under the preceptorship of Dr. Roger W. Pease, entering the classes of Syracuse University, and, as stated, receiving the degree of M. D. in 1877. He then went to Vienna and pursued a post-graduate course at the General Hospital. In 1878 he began practice in Syracuse, devoting himself largely to surgery and to laryngology in the beginning, but later relinquishing the latter branch. After having filled several successive positions in the University, he was elected to the chair of clinical surgery and laryngology in 1899, and until his death he was the senior surgical professor.

Of the many positions of honor that Dr. Jacobson has filled, of his numerous and valuable contributions to medical literature, of his professional ability and conscientious discharge of all duties, public and private, it is unnecessary to speak at length. To the younger generation, we wish to allude to him as a striking example of the falsity of the old contention that a medical prophet cannot achieve the highest honor in his own birth place. From a long, though necessarily occasional acquaintance, we can speak personally and with all sincerity of his greatness and goodness of heart, his integrity and industry.

## Figures and Tables

**Figure f1:**